# Supplementary figures and images for: Influence of tannic acid concentration on the physicochemical characteristics of saliva of spider monkeys (Ateles geoffroyi)
Source: PeerJ. 2022 Nov 25;10:e14402. doi: 10.7717/peerj.14402 (PMC9703984; doi:10.7717/peerj.14402)

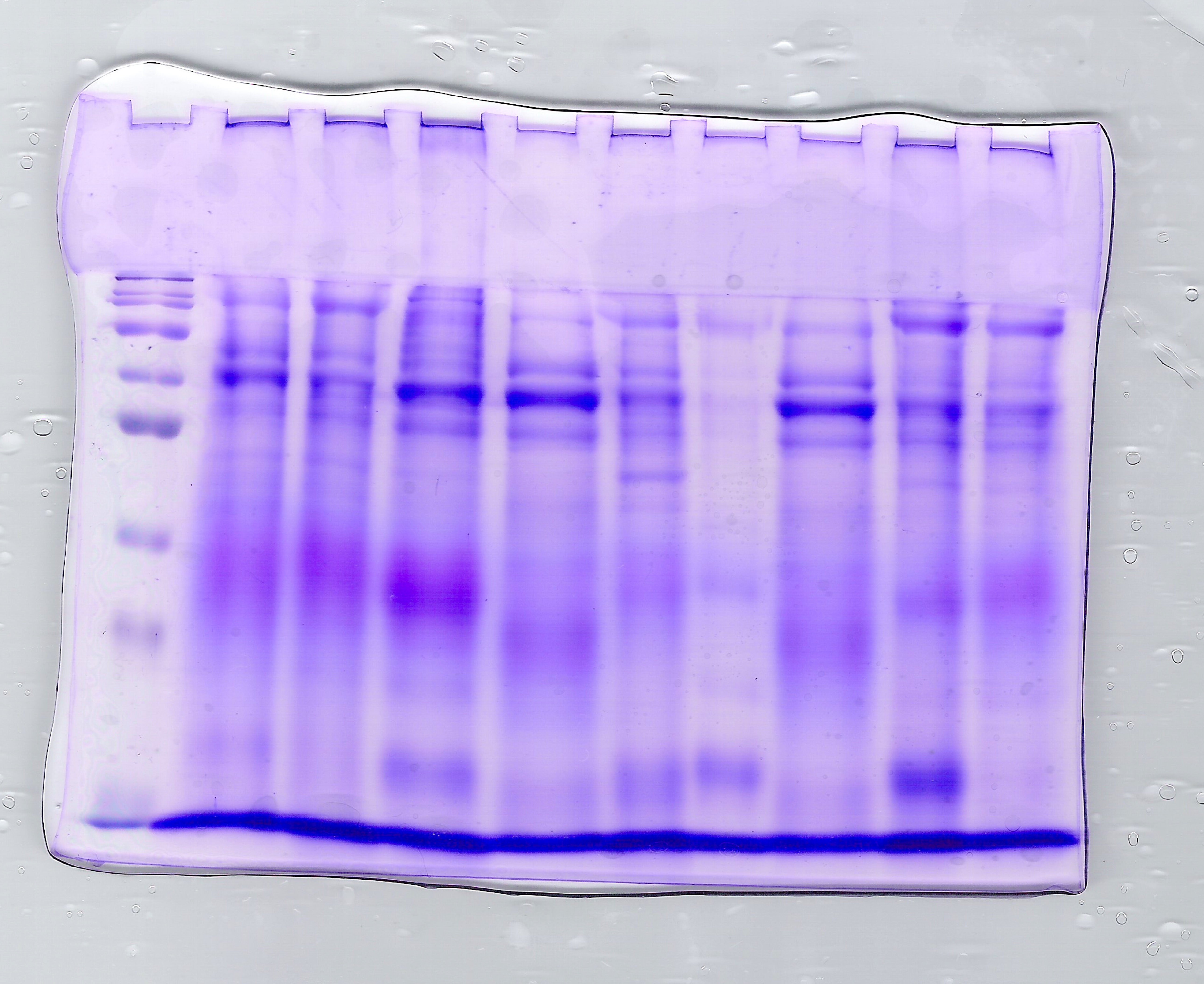

Supplement: Supplemental Information 1 — Control solution 0 mM, Tannin solution 0.01, 0.05, 0.1, 0.5 and 1 mM. [file peerj-10-14402-s001.zip › geles/0.01mm.jpg]

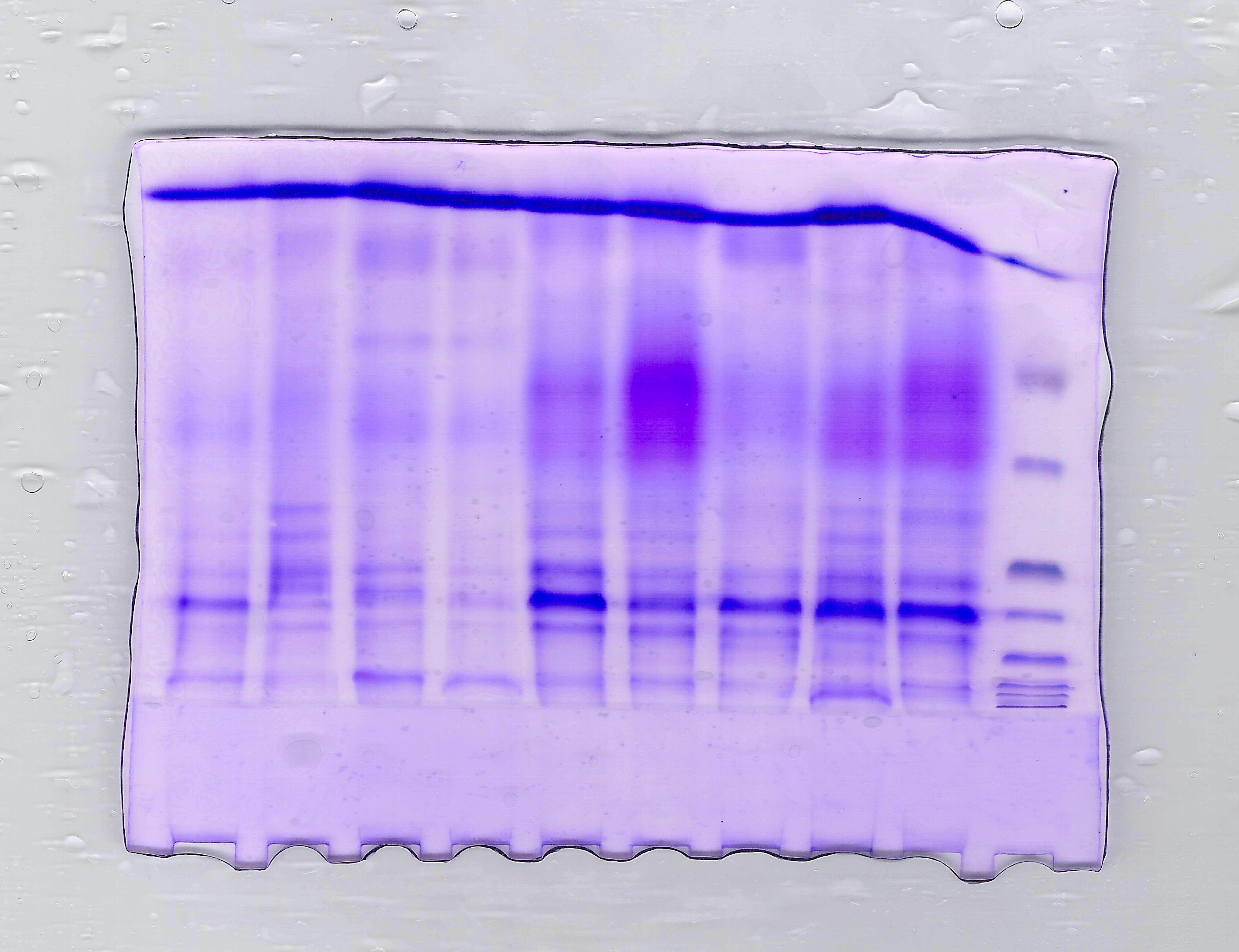

Supplement: Supplemental Information 1 — Control solution 0 mM, Tannin solution 0.01, 0.05, 0.1, 0.5 and 1 mM. [file peerj-10-14402-s001.zip › geles/0.05mm.jpg]

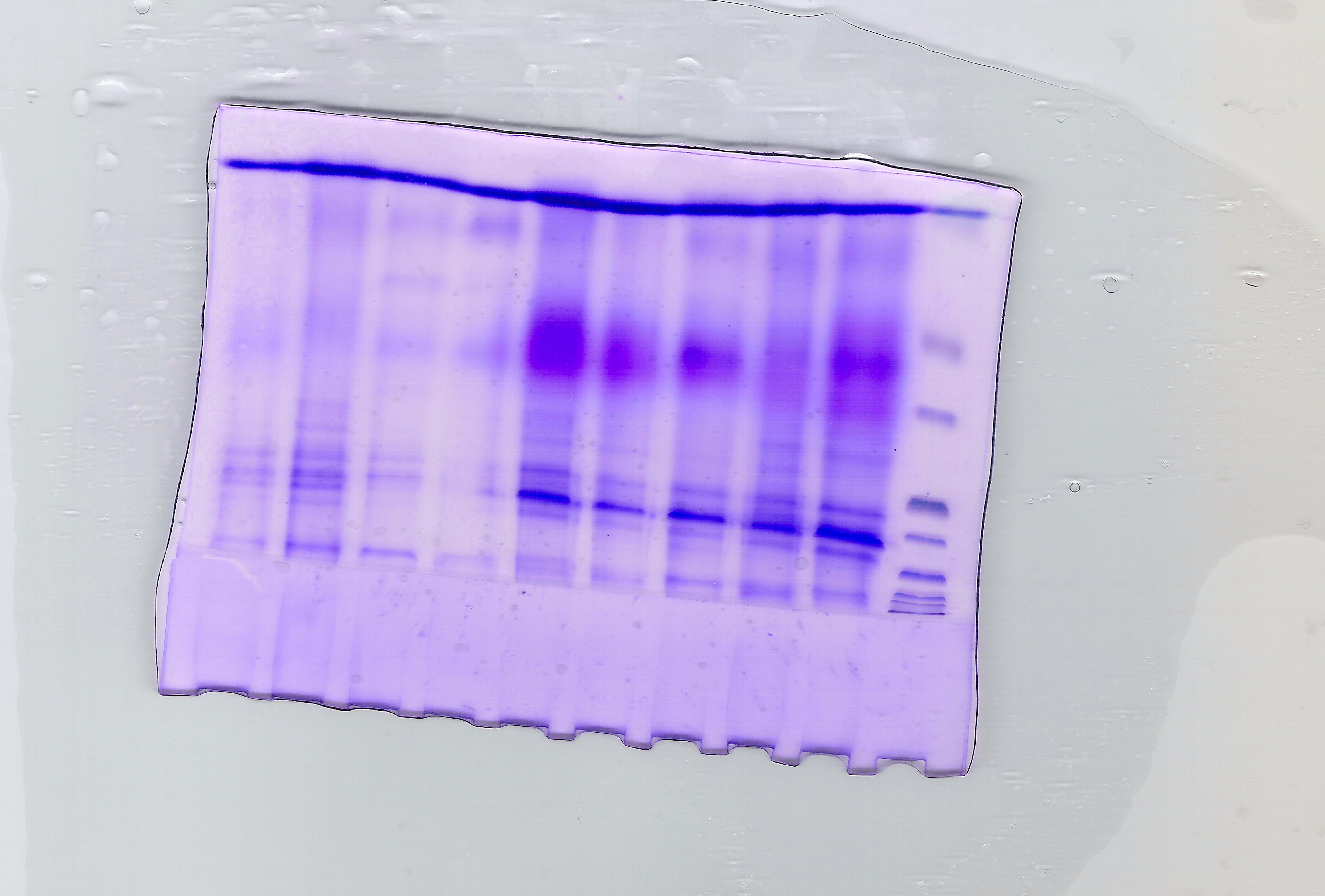

Supplement: Supplemental Information 1 — Control solution 0 mM, Tannin solution 0.01, 0.05, 0.1, 0.5 and 1 mM. [file peerj-10-14402-s001.zip › geles/0.1mm.jpg]

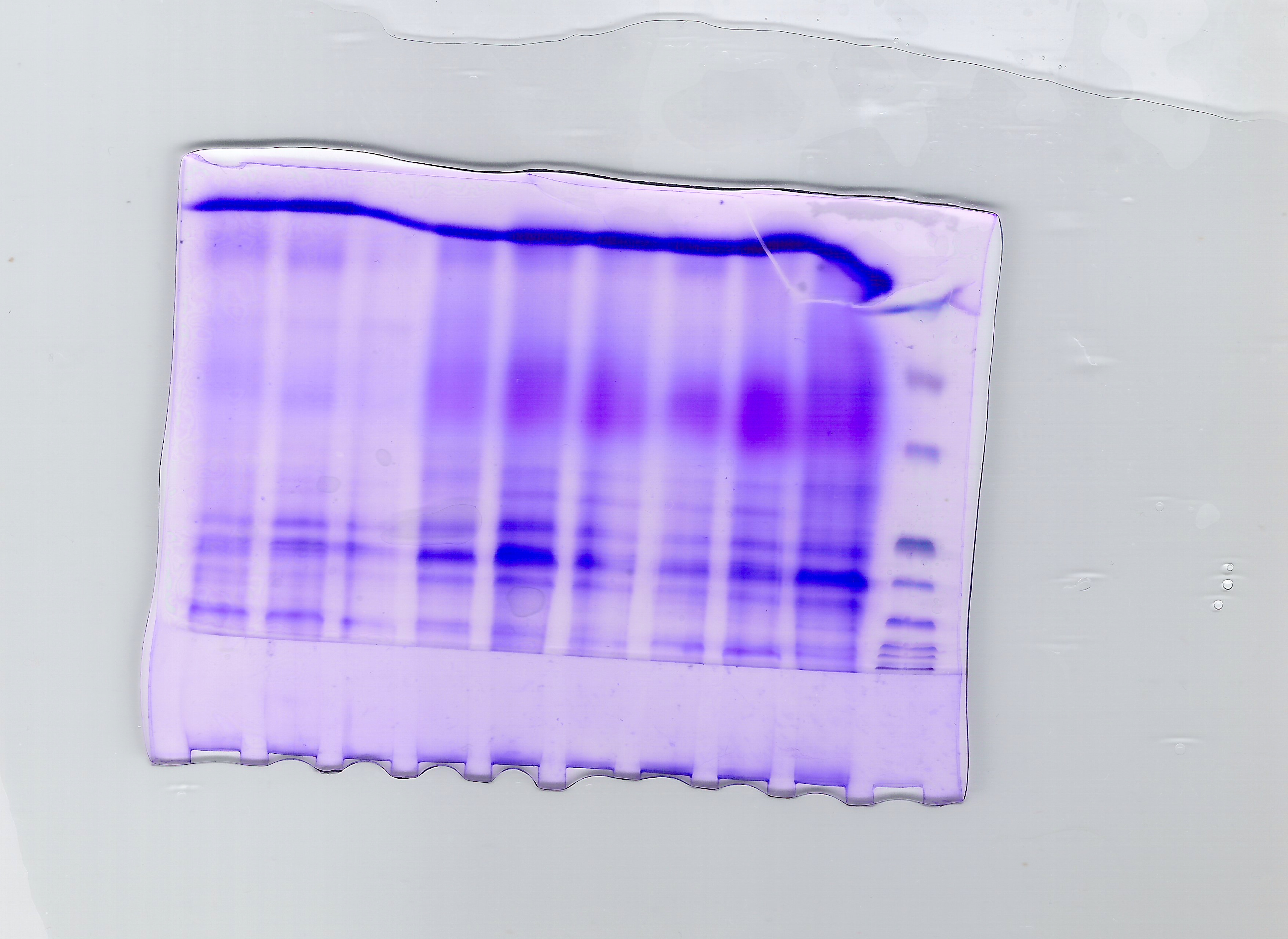

Supplement: Supplemental Information 1 — Control solution 0 mM, Tannin solution 0.01, 0.05, 0.1, 0.5 and 1 mM. [file peerj-10-14402-s001.zip › geles/0.5mm.jpg]

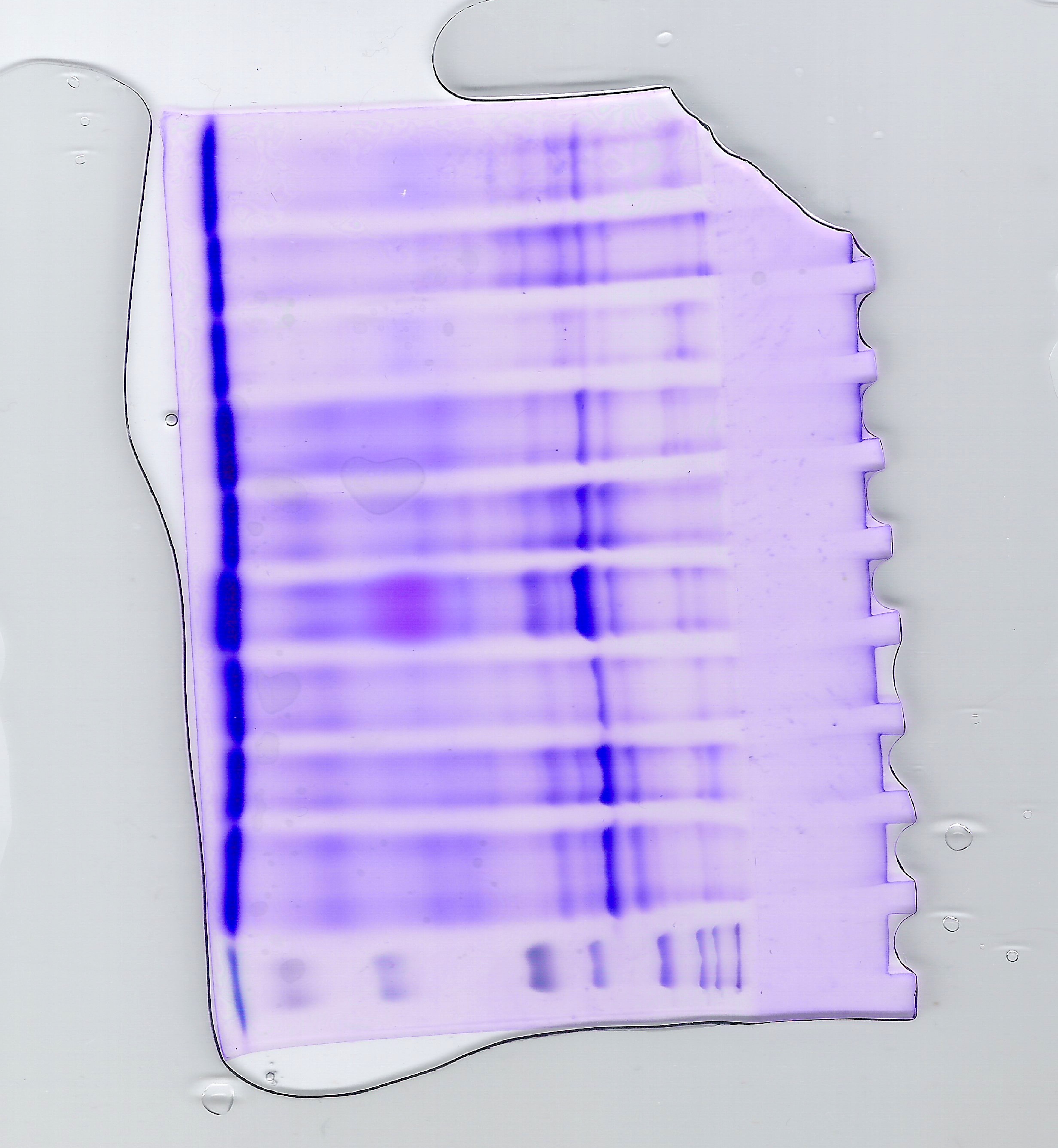

Supplement: Supplemental Information 1 — Control solution 0 mM, Tannin solution 0.01, 0.05, 0.1, 0.5 and 1 mM. [file peerj-10-14402-s001.zip › geles/0mm.jpg]

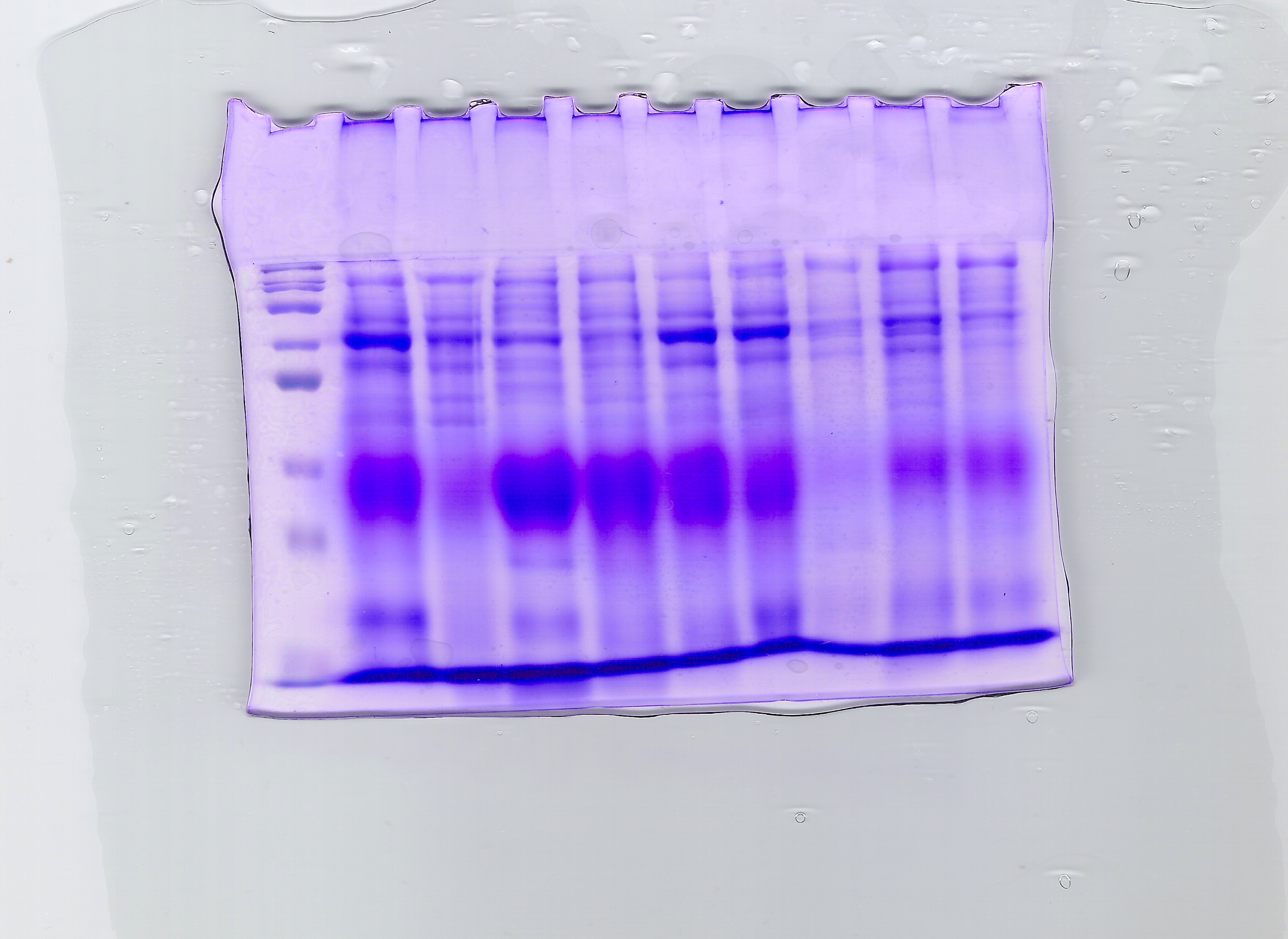

Supplement: Supplemental Information 1 — Control solution 0 mM, Tannin solution 0.01, 0.05, 0.1, 0.5 and 1 mM. [file peerj-10-14402-s001.zip › geles/1mm.jpg]
